# Transcriptomics combined with physiological analysis reveals the mechanism of cadmium uptake and tolerance in *Ligusticum chuanxiong* Hort. under cadmium treatment

**DOI:** 10.3389/fpls.2023.1263981

**Published:** 2023-09-22

**Authors:** Zhanling Zhang, Lele Zhong, Wanting Xiao, Yaping Du, Guiqi Han, Zhuyun Yan, Dongmei He, Chuan Zheng

**Affiliations:** ^1^ School of Pharmacy, Chengdu University of Traditional Chinese Medicine, Chengdu, Sichuan, China; ^2^ State Key Laboratory of Characteristic Chinese Medicine Resources in Southwest China, Chengdu University of Traditional Chinese Medicine, Chengdu, Sichuan, China; ^3^ Evaluation and Utilization of Strategic Rare Metals and Rare Earth Resource Key Laboratory of Sichuan Province, Chengdu, Sichuan, China; ^4^ Chengdu Analytical & Testing Center, Sichuan Bureau of Geology & Mineral Resources, Chengdu, Sichuan, China; ^5^ College of Medical Technology, Chengdu University of Traditional Chinese Medicine, Chengdu, Sichuan, China

**Keywords:** *Ligusticum chuanxiong* Hort., cadmium stress, subcellular localization, antioxidant system, transcriptomics

## Abstract

**Introduction:**

*Ligusticum chuanxiong* Hort. is a widely used medicinal plant, but its growth and quality can be negatively affected by contamination with the heavy metal cadmium (Cd). Despite the importance of understanding how *L. chuanxiong* responds to Cd stress, but little is currently known about the underlying mechanisms.

**Methods:**

To address this gap, we conducted physiological and transcriptomic analyses on *L. chuanxiong* plants treated with different concentrations of Cd^2+^ (0 mg·L^−1^, 5 mg·L^−1^, 10 mg·L^−1^, 20 mg·L^−1^, and 40 mg·L^−1^).

**Results:**

Our findings revealed that Cd stress inhibited biomass accumulation and root development while activating the antioxidant system in *L. chuanxiong*. Root tissues were the primary accumulation site for Cd in this plant species, with Cd being predominantly distributed in the soluble fraction and cell wall. Transcriptomic analysis demonstrated the downregulation of differential genes involved in photosynthetic pathways under Cd stress. Conversely, the plant hormone signaling pathway and the antioxidant system exhibited positive responses to Cd regulation. Additionally, the expression of differential genes related to cell wall modification was upregulated, indicating potential enhancements in the root cell wall’s ability to sequester Cd. Several differential genes associated with metal transport proteins were also affected by Cd stress, with ATPases, MSR2, and HAM3 playing significant roles in Cd passage from the apoplast to the cell membrane. Furthermore, ABC transport proteins were found to be key players in the intravesicular compartmentalization and efflux of Cd.

**Discussion:**

In conclusion, our study provides preliminary insights into the mechanisms underlying Cd accumulation and tolerance in *L. chuanxiong*, leveraging both physiological and transcriptomic approaches. The decrease in photosynthetic capacity and the regulation of plant hormone levels appear to be major factors contributing to growth inhibition in response to Cd stress. Moreover, the upregulation of differential genes involved in cell wall modification suggests a potential mechanism for enhancing root cell wall capabilities in isolating and sequestering Cd. The involvement of specific metal transport proteins further highlights their importance in Cd movement within the plant.

## 1 Introduction

In recent years, the pollution of soil by Cd has become a growing concern due to various sources such as metal smelting, sewage treatment, phosphorus fertilizer application, and atmospheric sedimentation (Huang et al., 2019b; Du et al., 2020; Ren et al., 2022). Cd is a toxic heavy metal, and excessive accumulation of Cd in the human body can cause damage to bones, kidneys, and lungs, resulting in long-term negative effects on human health (Staessen et al., 1999; Godt et al., 2006; Nawrot et al., 2006; Johri et al., 2010). *Ligusticum chuanxiong* Hort. belongs to the genus *Ligusticum* in the family Umbelliferae and is a famous medicinal plant. The dried rhizome of *L. chuanxiong* is widely recognized for its medicinal properties, especially its effectiveness in the treatment of cardiovascular and cerebrovascular diseases (Chen et al., 2018). It has been observed in several studies that traditional growing areas of *L. chuanxiong* have high levels of Cd in the soil (Qi-qua, 2014; Li et al., 2017; Deng et al., 2019). The excessive presence of Cd in the soil inhibits the growth of *L. chuanxiong* and leads to excessive accumulation of Cd in *L. chuanxiong*. This limits the development of the *L. chuanxiong* industry and threatens human health (Zeng et al., 2020; Xiao et al., 2021). Considering the detrimental effects of Cd and its impact on *L. chuanxiong*, it is essential to conduct research on Cd stress to uncover the mechanisms of Cd accumulation and tolerance in *L. chuanxiong* for future implications.

Plant growth under Cd stress is commonly associated with various detrimental effects, such as stunted growth, reduced lateral root development, decreased root vigor, and impaired water uptake and nutrient utilization (Hasan et al., 2009). Moreover, Cd accumulation in the above-ground parts of plants also leads to plant dwarfing, chlorosis, reduced biomass, and inhibition of photosynthesis (Liu et al., 2010). To reduce the toxic effects of Cd, plants have evolved a series of detoxification strategies. The cell wall is an important line of defense for Cd entry into plant cells (Tenhaken, 2014). For instance, under Cd stress, pectin in the cell wall can form chelates with Cd, while the cell wall cellulose content increases to limit the diffusion of Cd. Additionally, the lignin content of the secondary wall increases, thereby strengthening the cell wall and enhancing the plant’s tolerance to Cd (Liu et al., 2007; Ma et al., 2014). The Cd that enters the apoplast can subsequently move into the cytoplasm through cation exchange across the plasma membrane. Notably, ATPases, COPT-type, ZIP, and other metal transport proteins play crucial roles in Cd transport during this stage (Li et al., 2018b; Li et al., 2021). Some amino acids and organic acids are also involved in Cd transport, such as nicotinamide, histidine, malic acid, citric acid, etc. (Pilon et al., 2009). Substances such as reduced glutathione (GSH), phytochelatins (PCs), and metallothioneins in the cytoplasm chelate with Cd^2+^ and further translocate it to the vesicles for sequestration via the ABC transporter protein family. In addition, Cd stress induces a large amount of reactive oxygen species (ROS) production in plants, causing oxidative damage. At this time, the activities of antioxidative enzymes such as catalase (CAT), peroxidase (POD), and superoxide dismutase (SOD) will increase in plants to scavenge excess ROS from cells and reduce oxidative damage (Rizwan et al., 2016; Jiang et al., 2020). Overall, under Cd stress, plants employ various mechanisms, including cell wall fortification, enhanced Cd transport, chelation, and antioxidant defense activation, to alleviate the toxic effects and ensure their survival and growth.

With the rapid development of RNA sequencing (RNA-Seq) technology, RNA-Seq has been extensively utilized to investigate the molecular mechanisms of medicinal plants in response to different stresses (Li et al., 2018a; Zhao et al., 2019a; Wang et al., 2020). Nowadays, *Dendrobium officinale* (Jiang et al., 2020), *Verbena bonariensis* (Wang et al., 2019b), *Mulberry* (Dai et al., 2020), *Trifolium repens* (Wu et al., 2022), *Calotropis gigantea* (Yang et al., 2022), and *Hibiscus cannabinus* L. (Chen et al., 2021b), among other medicinal plants, are being investigated for their molecular mechanisms in response to Cd stress (Fan et al., 2023). For example, Chen et al. (2021b) found that after 20 days of Cd treatment in *mulberry seedlings*, the expression of RNA regulation (MYB, bHLH, and WRKY transcription factors), abiotic stress (genes encoding heat-shock protein), and signaling pathways (calmodulin-binding transcriptional activators, the protein kinase family proteins, and G-proteins) was upregulated, while the expression of secondary metabolism (genes related to the metabolism of flavonoids, phenylpropanoids, and terpenoid), hormone regulation (genes for the biosynthesis of jasmonates, ethylene and auxin), and the cell wall pathway (genes related to the synthesis, modification, and degradation of the cell wall) were downregulated. Wu et al. (2022) analyzed *Dendrobium*’s differential genes associated with Cd uptake, transport, and detoxification metabolic pathways. Metal transporters, sulfate glutathione metabolism, cell wall metabolism, and phenylpropanoid metabolism have important effects on adaptation to Cd stress. However, the molecular mechanisms of Cd stress response in medicinal plants are complex, and different medicinal plants may exhibit diverse Cd stress response mechanisms. At present, there are few reports on the molecular mechanisms of Cd stress response in *L. chuanxiong.*

In this study, we compared the integrated changes in the leaves and roots of *L. chuanxiong* under Cd stress by analyzing morphological characteristics and physiological indices. The transcriptome analysis of the roots of *L. chuanxiong* seedlings under Cd stress was performed using the RNA-Seq technique. The objectives of this study were as follows: (1) to investigate the changes in morphological characteristics and physiological responses of *L. chuanxiong* under Cd stress and (2) to screen key differentially expressed genes that respond to Cd treatment and preliminarily reveal the molecular mechanism of Cd accumulation and Cd tolerance in *L. chuanxiong*.

## 2 Materials and methods

### 2.1 Preparation of plant material and Cd treatment

Seedlings of *L. chuanxiong* were cultivated at the Pharmacy College, Chengdu University of Traditional Chinese Medicine, located in Sichuan Province, China (30° 41′ N, 104° 48′ E). Cluster buds of *L. chuanxiong* derived from plant tissue culture were planted in glass bottles with a volume of approximately 470 cm^3^. Each bottle contained 50 mL of 1/2 MS medium (1 mg·L^−1^ IBA, 8 g·L^−1^ agar, and 30 g·L^−1^ sucrose; pH 5.85). The seedlings were rooted in a culture chamber with controlled conditions, including a 14/10-h light/dark cycle, 65% humidity, and a temperature of 23°C ± 1°C. After 20 days, uniform and rooted *L. chuanxiong* seedlings weighing approximately 0.5 g each were selected. They were then transferred to glass bottles containing various concentrations of CdCl_2_·2.5H_2_O and 1/2 MS medium (1/2 MS, 8 g·L^−1^ agar, and 30 g·L^−1^ sucrose; pH 5.85) for the Cd stress treatment. The Cd^2+^ concentrations used were 5 mg·L^−1^ (Cd 5), 10 mg·L^−1^ (Cd 10), 20 mg·L^−1^ (Cd 20), and 40 mg·L^−1^ (Cd 40), while a control group (CK) without any Cd^2+^ was included (Cd^2+^ concentration of 0 mg·L^−1^). The culture chamber conditions remained constant throughout the experiment. Each experimental group consisted of 25 *L. chuanxiong* seedlings, and after 20 days of Cd stress treatment, samples were collected and processed for further analysis.

### 2.2 Measurement of growth indicators and sample processing

After 20 days of Cd stress treatment, *L. chuanxiong* plant samples from each experimental group were harvested. The samples were thoroughly rinsed with running water, followed by several rinses with distilled water. The excess surface water was removed using sterile blotting paper. The height and fresh weight of the *L. chuanxiong* seedlings were then measured. Subsequently, the root system was scanned using a root scanner (ScanMaker i800 Plus, Shanghai Microtek Technology Co., Ltd., Shanghai, China). The resulting images were analyzed using the Plant Root Analysis System (LA-S, Hangzhou WSEEN Testing Technology Co., Hangzhou, China) to assess parameters such as total root length, root surface area, root volume, number of root tips, and average root diameter of *L. chuanxiong*. Samples of *L. chuanxiong* roots and leaves for physiological index and subcellular fraction determination of Cd and *L. chuanxiong* roots for gene expression analysis were immediately frozen in liquid nitrogen and stored at −80°C in the refrigerator. All samples for total Cd content determination were dried in an oven at 60°C for 2 days, crushed, passed through a 0.3- mm mesh sieve, and then stored in a dry place for further estimations. Three biological replicates were set up for all experiments.

### 2.3 Determination of physiological indicators

The chlorophyll content in the leaves of *L. chuanxiong* was quantified following the method described by Rajalakshmi and Banu (2015). The activities of SOD, POD, CAT, and glutathione (GSH), as well as the content of malondialdehyde (MDA), were determined using commercially available kits from Suzhou Cominbio Biotechnology Co. Ltd., Suzhou, China. Each assay was performed according to the manufacturer’s instructions, and the absorbance was measured using a microplate reader (SpectraMax ABS PLUS, Molecular Devices Shanghai Co., Ltd., Shanghai, China). Activity and content calculations were carried out based on the measured absorbance values. We have shown detailed calculations and units of measure in the **Supplementary Documentation** (Word S1). Statistical analysis of the experimental data was conducted using Excel 2019 and SPSS 17.0, and differences between groups were assessed using the least significant difference (LSD) method with a significance level set at *p* < 0.05.

### 2.4 Determination of total Cd and subcellular distribution of Cd

Dried powders of *L. chuanxiong* roots and leaves (0.5 g for each sample) were digested using an electric hot plate at 170°C in a mixture of 7 mL of HNO_3_ (CAS 7697-37-2, 99%) and 1 mL of HClO_4_ (CAS 7601-90-3, 99%) (Schützendübel et al., 2001). The digestion process was carried out until complete digestion was achieved. Subsequently, the digested sample was diluted to a final volume of 25 mL with deionized water. The content of Cd in the *L. chuanxiong* samples was determined using ICP-MS (NexION, 2000, PerkinElmer Inc., Massachusetts, USA). The enrichment factor (EF) was equal to the ratio of the Cd content in the roots to the Cd content in 1/2 MS medium. The translocation factor (TF) was the ratio of Cd content in leaves to Cd content in roots.

The method of Weigel and Jäger (1980) was used to determine the subcellular distribution of Cd in *L. chuanxiong* with a slight improvement. The fresh sample of 0.5 g was ground with prechilled homogenate [250 mmol·L^−1^ of sucrose, 50 mmol·L^−1^ of Tris-HCL (pH 7.5), and 1 mmol·L^−1^ of dithioerythritol (C_4_H_10_O_2_S_2_)] at 4°C according to the ratio of material to liquid 1:10. The homogenates were sequentially centrifuged at 1,000 × *g* for 10 min and 10,000 × *g* for 30 min. The precipitates obtained were cell wall and organelle fractions, and the supernatants were cell-soluble components (including vacuole and ribosomes and proteins, etc.). The precipitates obtained from each component were digested using the same digestion method described above to determine the total Cd content. The supernatant was diluted with deionized water and directly used for Cd content determination.

### 2.5 RNA extraction, library preparation, and sequencing

Total RNA was extracted from the tissue using TRIzol® Reagent (Invitrogen Corporation, California, USA), and genomic DNA was removed using DNaseI (Takara Bio Inc., Shiga, Japan) (Pandey et al., 2022). Then the integrity and purity of the total RNA quality were determined by the 2100 Bioanalyser (Agilent Technologies, Inc., California, USA) and quantified using the ND-2000 (NanoDrop Technologies, Inc., Delaware, USA).

RNA purification, reverse transcription, library construction, and sequencing were performed at Shanghai Majorbio Bio-pharm Biotechnology Co. Ltd. (Shanghai, China). The transcriptome library was prepared following the TruSeqTM RNA sample preparation Kit from Illumina (San Diego, CA, USA) using 1 μg of total RNA. Shortly, messenger RNA was isolated according to the polyA selection method by oligo (dT) beads and then fragmented by fragmentation buffer first. Secondly, double-stranded cDNA was synthesized using a SuperScript double-stranded cDNA synthesis kit (Invitrogen, CA) with random Hexamer primers (Illumina). The synthesized cDNA was then subjected to end-repair, phosphorylation, and “A” base addition according to Illumina’s library construction protocol. Libraries were size selected for cDNA target fragments of 300 bp on 2% Low Range Ultra Agarose, followed by PCR amplification using Phusion DNA polymerase (NEB) for 15 PCR cycles. After quantified by TBS380, the paired-end RNA-seq library was sequenced with the Illumina NovaSeq 6000 sequencer (2 bp × 150 bp read length).

### 2.6 *De novo* assembly and annotation

The raw paired-end reads were trimmed, and quality was controlled by FastP with default parameters. Subsequently, clean data from the samples were used to do *de novo* assembly with Trinity. The assembled transcripts were then assessed and optimized with BUSCO, TransRate, and CD-HIT. All the assembled transcripts were searched against the NCBI protein nonredundant (NR), Swiss-Prot, Pfam, Clusters of Orthologous Groups of proteins (COG), GO, and KEGG databases using BLASTX to identify the proteins that had the highest sequence similarity with the given transcripts to retrieve their function annotations, and a typical cutoff *E*-values of less than 1.0 × 10^−5^ was set.

### 2.7 Differential expression analysis and functional enrichment

To identify differentially expressed genes (DEGs) between two different groups, the expression level of each gene was calculated according to the transcripts per million reads (TPM) method. RSEM was used to quantify gene abundances (Smyth, 2005; Li and Dewey, 2011). Essentially, differential expression analysis was performed using the DEGseq2, DEGs with |log2 (foldchange)|≥1, and *p*-adjust ≤ 0.05 were considered to be significantly differentially expressed genes. In addition, functional-enrichment analysis including GO and KEGG was performed to identify which DEGs were significantly enriched in GO terms and metabolic pathways at *p*-adjust ≤ 0.05 compared with the whole-transcriptome background. GO functional enrichment and KEGG pathway analysis were carried out by Goatools (https://github.com/tanghaibao/Goatools) and KOBAS (http://kobas.cbi.pku.edu.cn/home.do) (Xie et al., 2011).

### 2.8 qRT-PCR validation

Roots of *L. chuanxiong* exposed to 20 mg·L^−1^ and 40 mg·L^−1^ Cd for 20 days were used in qRT-PCR. A total of 10 random DEGs were selected for qRT-PCR analysis, with each gene being analyzed with three replicates. The RNA used for qRT-PCR analysis was isolated from the same RNA sequencing samples mentioned earlier. qRT-PCR was performed on a Fluorescent Quantitative PCR Detection system (LineGene 4800, BIOER, Hangzhou, China) using SYBR-GREEN1 fluorescent reagents (Foregene, Chengdu, China). Thermocycler settings were as follows: 95°C for 3 min, 40 cycles of 95°C for 15 s, 60°C for 30 s, and 72°C for 10 s. In addition, melting curves from 65°C to 95°C were used to confirm reaction specificity. All primers are listed in **Supplementary Table S1**. Actin served as a normalization control. The 2^−ΔΔCt^ method was used to calculate relative gene expression.

## 3 Results

### 3.1 Changes in morphological characteristics of *L. chuanxiong* under Cd treatment

**Figure 1:**
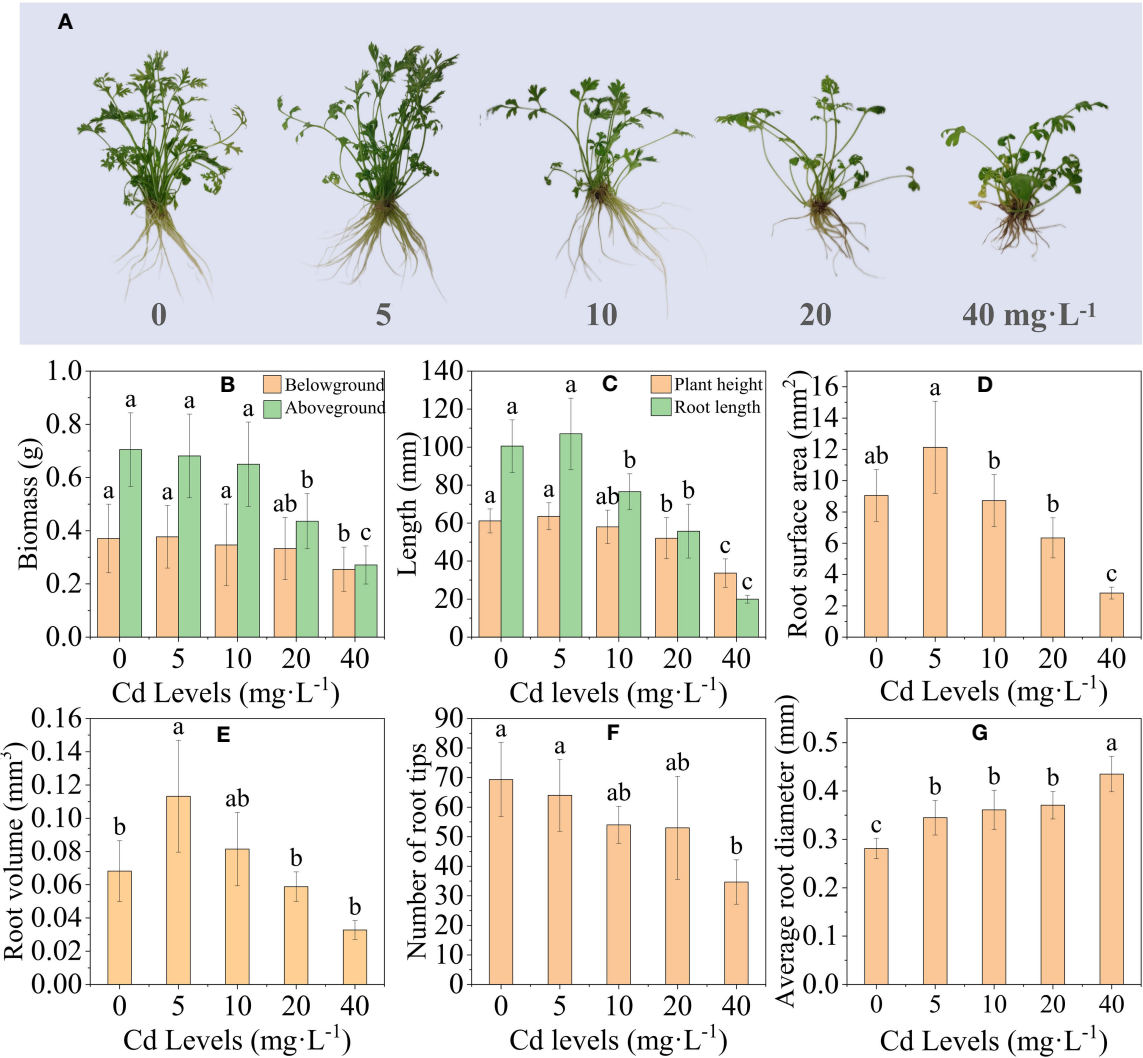
Effect of different concentrations of Cd treatment on the growth of *L. chuanxiong.* **(A)** Growth of *L. chuanxiong* under different concentrations of Cd treatment; **(B)** changes in biomass of *L. chuanxiong*; **(C)** plant height and total root length; **(D)** root surface area; **(E–G)** root volume, root tip number and mean root diameter, in that order. Bars represent the mean ± standard deviation (*n* = 3). Values marked with different letters differ significantly (*p* < 0.05).

Compared to the control group (CK), the *L. chuanxiong* plants treated with 5 mg·L^−1^ and 10 mg·L^−1^ showed no apparent signs of toxicity. However, the plants treated with 20 mg·L^−1^ and 40 mg·L^−1^ exhibited clear symptoms of toxicity, including yellowing and wilting (**Figure 1A**). Except for Cd 5, the height, total root length, and fresh weight of the aboveground and belowground parts of the *L. chuanxiong* plant were inhibited to different degrees with increasing Cd concentration (**Figures 1B, C**). Compared to the CK group, the height of plants exposed to 40 mg·L^−1^ Cd treatment was significantly reduced from 55.87 mm to 38.53 mm, total root length was significantly reduced from 100.52 mm to 19.98 mm, and aboveground mass and belowground biomass were significantly reduced by 61.15% and 32.54%, respectively. The root scan results showed that the trends of root surface area, root volume, and root tip number were consistent with the above results except for the mean root diameter (**Figures 1D–G**). Cd stress significantly affected the growth and development of *L. chuanxiong*, and the high concentration of Cd treatment inhibited the growth and reduced the biomass accumulation of *L. chuanxiong.*

### 3.2 Effect of Cd treatment on the physiological indices of *L. chuanxiong*

**Figure 2:**
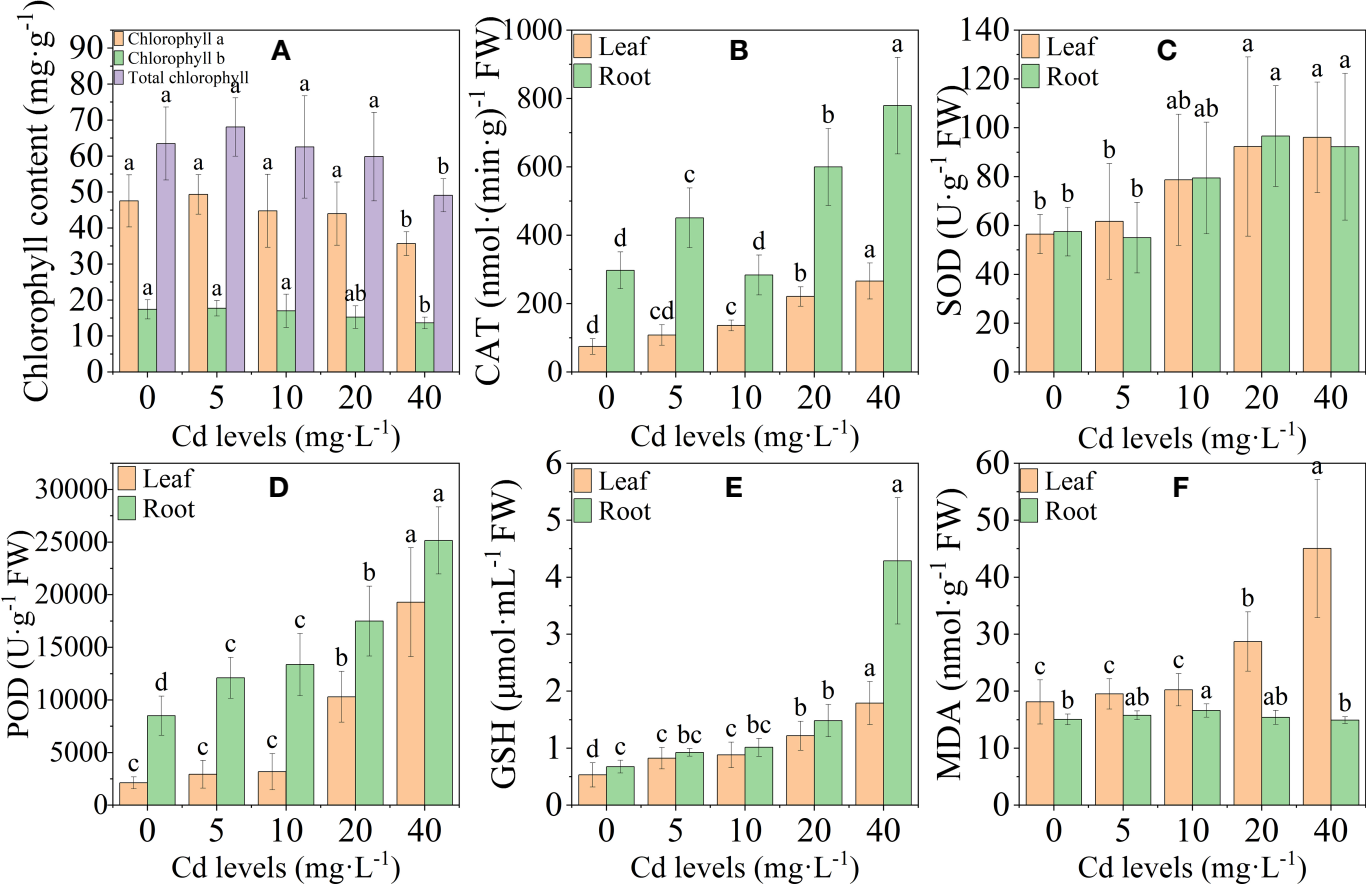
Changes in physiological indicators of chuanxiong after 20 days of treatment with different Cd concentrations. **(A)** Chlorophyll content; **(B)** CAT activity in leaves and roots; **(C–F)** indicate SOD, POD, GSH, and MDA, respectively. Bars represent the mean ± standard deviation (*n* = 3). Values marked with different letters are significantly different (*p* < 0.05). FW indicates fresh weight.

The chlorophyll content (chlorophyll *a*, chlorophyll *b*, and total chlorophyll) in the leaves of *L. chuanxiong* decreased gradually as the Cd concentration increased, and it was significantly lower than the control at the 40-mg·L^−1^ treatment (**Figure 2A**). In the plant’s antioxidant system, the activities of antioxidant enzymes CAT, SOD, and POD and nonenzymatic antioxidant GSH, as well as the MDA content in leaves, were positively regulated by Cd stress overall and reached a maximum at 40 mg·L^−1^ Cd treatment. The trends of CAT, SOD, POD, and GSH in roots were basically the same as those in leaves, and the MDA content reached a maximum at 10 mg·L^−1^ and then gradually decreased (**Figures 2B–F**). The high concentration of Cd treatment reduced chlorophyll accumulation in the leaves of *L. chuanxiong*, resulting in a decrease in the photosynthetic capacity of *L. chuanxiong* seedlings. Cd stress activated the antioxidant system of *L. chuanxiong*, and the significant increase in the activity of antioxidant substances was beneficial to alleviating the toxic effects of Cd.

### 3.3 Total and subcellular distribution of Cd

**Figure 3:**
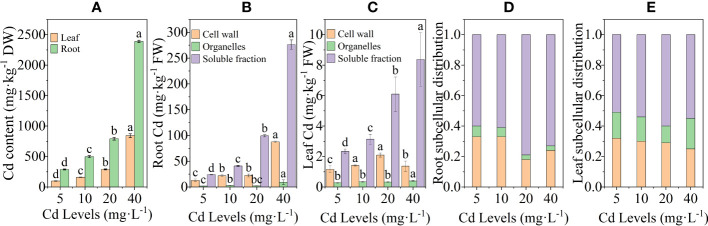
Cd content and subcellular distribution of Cd in roots and leaves after 20 days of treatment with different Cd concentrations. **(A)** Cd content in leaves and roots; **(B)** subcellular distribution of cadmium in roots; **(C)** subcellular distribution of cadmium in leaves; **(D, E)** percentage of Cd content of each fraction in roots and leaves. Bars represent mean ± standard deviation (*n* = 3). Values marked with different letters are significantly different (*p* < 0.05). DW, dry weight; FW, fresh weight.

The Cd content in the CK group was not measured due to being below the detection limit of the device, while the remaining groups showed a positive correlation with the Cd treatment concentration, both in roots and leaves. The Cd content in leaves and roots reached a maximum value of 843.22 mg·kg^−1^ and 2,389.83 mg·kg^−1^ at 40 mg·L^−1^ concentration, which was 757.98% and 730.06% higher compared to 5 mg·L^−1^, respectively (**Figure 3A**). The EF of *L. chuanxiong* gradually decreased with increasing Cd concentration and increased to 59.74 after reaching the lowest point of 39.62 at 20 mg·L^−1^ concentration treatment. The TF, on the other hand, showed no obvious trend and was stable between 0.32 and 0.36 (**Table 1**). The Cd content in the roots was significantly higher than that in the aboveground parts in all the different treatment groups, which showed that in *L. chuanxiong*, the Cd accumulated mainly in the roots. The enrichment and transfer coefficients of *L. chuanxiong* demonstrated its significant ability to enrich Cd in the growing environment and transfer it from the underground part to the aboveground part in a stable proportion.

**Table 1:** Enrichment factor and translocation factor of *L. chuanxiong* under different Cd concentration treatments.

Cd levels(mg·kg^-1^)		5	10	20	40
Factor	EF^1^	57.58	49.92	39.62	59.74
	TF^2^	0.34	0.32	0.36	0.35

EF^1^, enrichment factor; TF^2^, translocation factor. Values are the average of three biological replicates.

The results of the subcellular distribution of Cd in the roots and leaves of *L. chuanxiong* showed that the higher the concentration of Cd treatment, the greater the accumulation of Cd in the cell wall, organelle, and soluble fraction (**Figures 3B, C**). The shares of soluble fraction, cell wall fractions, and organelles in the roots were 60%–80%, 18%–33%, and 2%–5%, respectively. The shares in leaves were 62%–83%, 13%–31%, and 4%–7% (**Figures 3D, E**). Cd was mainly distributed in the cell-soluble fraction, followed by the cell wall, and the lowest percentage of Cd was found in the cell organelles. The first two contained up to 93%–98% of Cd in the whole cell, which was the main storage location of Cd in *L. chuanxiong* cells.

### 3.4 RNA-seq and annotation results

**Figure 4:**
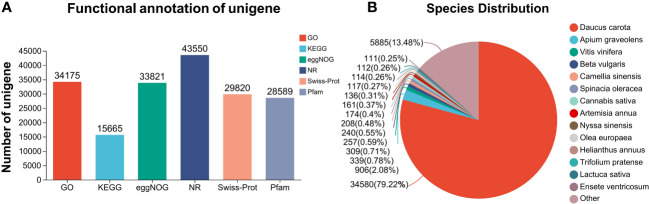
RNA-seq annotation results. **(A)** Number of unigenes successfully annotated to different databases; **(B)** species similarity.

Combined with the changes in phenological characteristics and physiological analysis of *L. chuanxiong* under Cd treatment, the growth of *L. chuanxiong* was significantly affected by Cd treatment under Cd 20 and Cd 40 treatments. Therefore, we selected three groups, CK, Cd 20, and Cd 40, of Cd-treated roots of *L. chuanxiong* for 20 days for transcriptome sequencing analysis. A total of nine cDNA libraries were constructed and sequenced from three independent biological replicates. More than 41.36 million (6.04 Gb) clean reads were obtained for each sample (**Supplementary Table S2**). The quality fraction of Q30 levels ranged from 92.42% to 93.61%, and the average GC content ranged from 42.56% to 43.08% (**Supplementary Table S2**). The average length was 980 BP (**Supplementary Table S2**). In comparison to the six publicly available databases, 90,335 annotated unigenes were obtained. The number of successfully annotated unigenes in the six databases is shown in **Figure 4A**. *Daucus carota* L. provided 79.22% similarity to *L. chuanxiong*, followed by *Apium graveolens* L. (2.08%) (**Figure 4B**). This indicates high sequencing quality and sufficient sequencing depth for transcriptome analysis. The raw data obtained are stored in the National Center for Biotechnology Information Biology Project (NCBI) database (access number PRJNA960886, access link: https://www.ncbi.nlm.nih.gov/sra/PRJNA960886).

### 3.5 Analysis of differentially expressed genes in GO term and KEGG metabolic pathways

**Figure 5:**
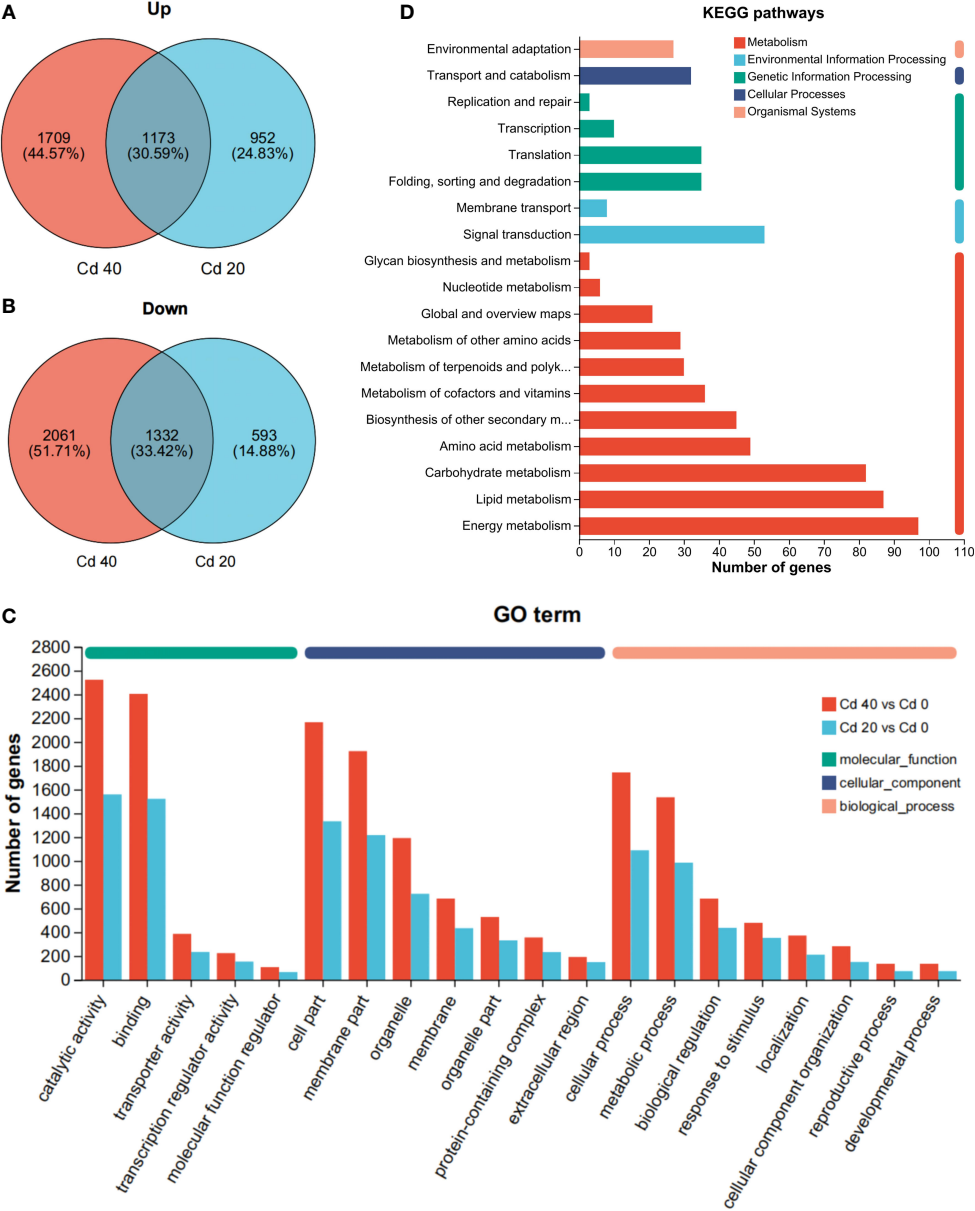
Annotation results of differentially expressed genes. **(A, B)** Venn analysis of differentially expressed genes: **(A)** upregulated; **(B)** downregulated. **(C)** Go term annotation results. **(D)** KEGG pathways annotation results.

To further understand the altered gene expression levels of *L. chuanxiong* in response to Cd stress, differential expression analysis was performed with DEGseq2. Compared with the control, 4,050 (2,125 upregulated, 1,925 downregulated) and 6,174 (2,861 upregulated, 3,313 downregulated) DEGs were obtained for Cd 20 and Cd 40, respectively. Both together had 1,173 upregulated and 1,332 downregulated differential genes (**Figures 5A, B**).

Cd 20 and Cd 40 are annotated to 48 and 49 GO terms, respectively. Among them, 21, 15, 12, and 21 GO terms belong to biological processes (BP), 15 to molecular functions (MF), and 13 to cellular components (CC) (**Supplementary Table S3**). Among the GO terms shared by both, catalytic activity and binding represent the most abundant categories of MF; cell part and membrane part appear more in the category of CC; and cellular processes and metabolic processes account for the largest proportion of BP (**Figure 5C**). KEGG analysis revealed 118 (1,094 DEGs) and 127 (1,929 DEGs) pathways, respectively (**Supplementary Table S4**). Compared with the control group, the common pathways of Cd 20 and Cd 40 are mainly concentrated in energy metabolism, lipid metabolism, carbohydrate metabolism, signal transduction, and amino acid metabolism (**Figure 5D**). Among them, differential expression of metabolic pathways such as biosynthesis of unsaturated fatty acids, MAPK signaling pathway —plant, and glutathione metabolism genes were mostly upregulated. Photosynthesis, plant hormone signal transduction, photosynthesis-antenna proteins, phenylpropanoid biosynthesis, and carbon fixation in photosynthetic organisms were mostly downregulated (**Supplementary Figure S5**).

### 3.6 DEGs and metabolic pathways associated with Cd transport and detoxification

All 42 DEGs of the photosynthetic pathway were significantly downregulated (**Supplementary Table S6**), mainly associated with photosystem I (20), photosystem II (9), and ferredoxin (5). There were 19 DEGs of the photosynthetic antenna protein, all significantly downregulated, of which 14 DEGs were associated with chlorophyll *a*– *b*-binding proteins (**Supplementary Table S6**). The carbon fixation in photosynthetic organisms (four upregulated, 14 downregulated) was dominated by DEGs such as glyceraldehyde-3-phosphate dehydrogenase (two upregulated, three downregulated), fructose-bisphosphate aldolase (two downregulated), fructose-1,6-bisphosphatase (two downregulated), phosphoenolpyruvate carboxylase (two downregulated), and ribulose diphosphate carboxylase (two downregulated) (**Supplementary Table S6**).

There were 35 DEGs in the antioxidant system of *L. chuanxiong* that were affected by Cd stress (14 upregulated, 21 downregulated), of which 28 DEGs were associated with POD (11 upregulated, 17 downregulated). In the glutathione metabolic pathway, there were 18 genes whose expression was regulated by Cd (13 upregulated and five downregulated). These included glutathione S-transferase (four upregulated, two downregulated), glutathione transferase (three upregulated), glutathione peroxidase (two upregulated), glutathione (one upregulated), glutathione reductase (one upregulated), etc. (**Supplementary Table S7**).

Auxin is an important plant growth regulator that can induce root growth by enhancing cell division and elongation. In the plant hormone signal transduction pathway, 15 DEGs associated with the auxin synthesis pathway were significantly downregulated in most DEGs, except for *SAUR* family genes. Ethylene is a simple and very important gaseous phytohormone, and six ethylene signaling pathway-related genes (*EBF1*, *ERF1*, *ETR*) were upregulated in expression under Cd stress. In addition, abscisic acid signaling pathway (*PYL*, *PP2C*) genes, salicylic acid transcription factor (*TGA2*), and gibberellin receptor (*GID1C*) were also regulated by Cd (**Supplementary Table S8**).

In the plant cell wall modification pathway, the DEGs of pectin esterase (seven upregulated, four downregulated) and cellulose synthase (one upregulated) are regulated by Cd. The expression levels of DEGs related to the lignin biosynthesis pathway, such as laccase (3), caffeic acid *O*-methyltransferase, bergaptol *O*-methyltransferase, and cinnamaldehyde dehydrogenase, were upregulated (**Supplementary Table S9**).

Among the metal transporter family, we annotated 11 *ABC* transporter proteins (nine upregulated and two downregulated), four calcium transporter proteins (*ATPase*, three upregulated), magnesium transporter protein (*MRS2*), and Cd/Zn transporter ATPase (*HAM3*)-related genes differentially expressed under Cd stress (**Supplementary Table S10**).

### 3.7 qRT-PCR validation

**Figure 6:**
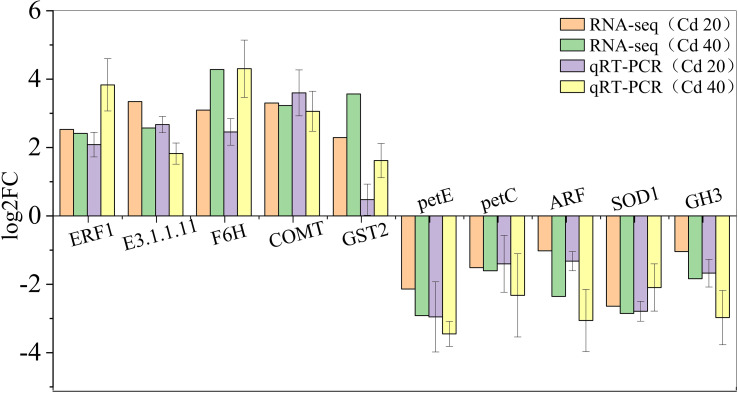
Validation of RNA-Seq results using qRT-PCR. Comparing the log2FC values of RNA-seq and qRT-PCR (log2FC > 0 indicates upregulation, log2FC < 0 indicates downregulation), if the expression trends of RNA-seq and qRT-PCR are consistent, it indicates that the results of RNA-seq are available (*n* = 3).

To validate the RNA-seq results, 10 DEGs were randomly selected for qRT-PCR analysis to determine the mRNA levels after exposure to 20 mg·L^−1^ and 40 mg·L^−1^ Cd^2+^. The results showed that the relative gene expression trends of qRT-PCR were consistent with RNA-seq, demonstrating the reliability of the RNA-seq data (**Figure 6**).

## 4 Discussion

### 4.1 Effect of Cd treatment on the growth index of *L. chuanxiong*

We evaluated the effect of Cd treatment at different concentrations on the growth of *L. chuanxiong*. Low concentrations of Cd treatment stimulated the growth of *L. chuanxiong*, and similar findings were found for plants such as Oxalis *Calotropis gigantea* L. (Yang et al., 2022) and *Lonicera japonica* Thunb. (Jia et al., 2015). As the concentration of Cd increased, so did the growth of *L. chuanxiong* seedlings, which was inhibited with a decrease in plant height and biomass. The 40-mg·L^−1^ Cd treatment resulted in a significant reduction in total root length, root surface area, root volume, and root tip number in *L. chuanxiong*, and similar results were found in studies on Cd treatment of *Solanum nigrum* L. (Guo et al., 2009). Previous studies have demonstrated that plants exhibit the highest efficiency in cation absorption near the root tips (Piñeros et al., 1998; Huang et al., 2019a; Rivetta et al., 2023). The reduction in the number of root tips and root surface area significantly diminishes the effective area of the plant root system for Cd uptake, ultimately determining its uptake capacity (Hasan et al., 2009). The inhibition of root system growth is a protective mechanism that prevents *L. chuanxiong* from excessive contact with Cd, thus minimizing its uptake and accumulation. This approach is advantageous in mitigating Cd toxicity and enhancing *L. chuanxiong*’s tolerance toward Cd. However, the inhibition of root elongation also hampers *L. chuanxiong*’s ability to absorb and utilize water and nutrients, consequently limiting its overall growth (Hasan et al., 2009).

### 4.2 Cd accumulation and subcellular distribution of Cd in *L. chuanxiong*

Except for a few hyperaccumulating plants, most plants store Cd mainly in the roots (Zacchini et al., 2009). As with many plants, more than 63% of Cd was concentrated mainly in the roots of *L. chuanxiong*. The Cd content of all parts of *L. chuanxiong* showed a linear increase with increasing Cd treatment concentration. The Cd enrichment factor in its roots fluctuated between 39.62 and 59.74, with the translocation factor less affected by Cd concentration. The roots of *L. chuanxiong* have a strong Cd enrichment capacity and are highly susceptible to enrichment from the survival environment to higher concentrations of Cd, which are transferred to the leaves in a certain proportion. The high accumulation characteristics of Cd in *L. chuanxiong*, coupled with the increasing Cd content in the soil of traditional cultivation areas, have contributed to the increasing Cd pollution problem of *L. chuanxiong* in recent years (Ma et al., 2014; Qi-qua, 2014; Li et al., 2017). At present, implementing soil ecological remediation in traditional planting areas or selecting alternative planting sites with lower Cd content may be an effective measure to solve the problem of Cd contamination in *L. chuanxiong*.

The subcellular distribution of heavy metals is closely related to the tolerance and detoxification of heavy metals in plants. Our research findings reveal that approximately 18% –33% of Cd is stored in the cell wall. The cell wall contains many hydroxyl, thiol, and carboxyl functional groups, which can form Cd complexes with Cd and prevent the flow of Cd to the protoplasts (Harholt et al., 2010). The study by Feng et al. (2018) found that when plants are subjected to Cd stress, their cell walls thicken, thereby binding more Cd, which is consistent with our study. More than 62% of Cd is stored in the cell- soluble fraction. Vacuoles are the main soluble fraction of cells. When the binding capacity of Cd on the cell wall reached saturation, a large amount of Cd passing through the cell wall was transported to the vacuole for sequestration (Mendoza-Cózatl et al., 2010). These findings indicate that in *L. chuanxiong*, Cd mainly accumulates in the roots through vacuole compartmentalization and fixation within the cell wall. This strategy reduces the transfer of Cd to the aboveground parts of the plant, ensuring their growth and development, thereby serving as an important tolerance mechanism for *L. chuanxiong.*

### 4.3 Effect of Cd treatment on photosynthesis of *L. chuanxiong* seedlings

During photosynthesis in plants, chlorophyll plays a crucial role in the absorption and utilization of light energy. A high concentration of Cd significantly inhibited chlorophyll accumulation in the *L. chuanxiong* leaves. The reduction of chlorophyll content will affect the plant’s photosynthetic efficiency and lead to the failure of the plant to grow normally. Transcriptome sequencing results showed that most of the DEGs in photosynthesis, photosynthesis-antenna proteins, and carbon fixation in photosynthetic organism pathways were significantly downregulated under Cd stress treatment. Cd stress inhibited light energy uptake, electron transfer, and the Calvin cycle from disrupting photosynthesis in *L. chuanxiong*, resulting in a decrease in photosynthetic capacity and growth inhibition in *L. chuanxiong.* Similar findings were observed in a study on cotton (Farooq et al., 2016).

### 4.4 Cd treatment activates the antioxidant system of *L. chuanxiong*

Cd stress leads to the production of a large amount of reactive oxygen species (ROS) in plants and causes oxidative damage (Nagajyoti et al., 2010; Ehsan et al., 2014). Plants possess antioxidative enzymes (POD, SOD, CAT) that can convert toxic H_2_O_2_ and ${\text{O}}_{2}^{\;-1}$ into harmless oxygen and water, effectively scavenging ROS (Haider et al., 2021). In our study, we observed that the activities of antioxidative enzymes (CAT, POD, SOD) in *L. chuanxiong* increased gradually with higher concentrations of Cd treatment. Additionally, we identified several differentially expressed genes related to the antioxidant system in *L. chuanxiong* that responded positively to Cd stress. This suggests that the antioxidant system of *L. chuanxiong* plays an important role in scavenging ROS and resisting oxidative damage caused by Cd stress. MDA is one of the breakdown products of polyunsaturated fatty acids in membranes and is commonly used to evaluate the degree of oxidative damage (Demiral and Türkan, 2005). In our study, we observed a gradual increase in MDA content in the leaves of *L. chuanxiong*, while it remained relatively stable in the roots. This suggests that the root system has a stronger capacity to resist oxidative damage compared to the leaves. GSH, as a nonenzymatic antioxidant, also plays an important role in the plant antioxidant system. On the one hand, it can directly reduce ROS and alleviate Cd-induced oxidative stress. On the other hand, Cd ions can also form complexes with GSH and then translocate to the vacuole or apoplast for detoxification (Yadav, 2010). The subcellular distribution of Cd in *L. chuanxiong* showed that Cd is mainly distributed in the soluble components of cells, which may be related to the chelation and transport of GSH. We annotated in our RNA-seq results that several DEGs (*GST* and *GPX*, etc.) of the glutathione pathway are upregulated. Some of the GST genes have been shown to have enhanced Cd tolerance and reduced Cd accumulation in plants (Zhao et al., 2009; Dixit et al., 2011; Wu et al., 2019).

### 4.5 Plant hormone signal transduction pathway positively responds to Cd treatment and may affect the growth and Cd accumulation of *L. chuanxiong*

Plant hormone signaling pathways (IAA, ethylene, SA, GA, JA, and ABA) were found to respond positively to Cd regulation in the present study. Under Cd stress, changes in plant hormone levels affect plant growth and development and mediate the accumulation of Cd in plants (Qin and Huang, 2018; Nguyen et al., 2021). Cd stress resulted in significant down regulation of gene expression levels of the *GH3*, *AUX/IAA*, and *AFR* families in the IAA biosynthetic pathway of *L. chuanxiong.* Cd severely disrupts the synthesis and transport of IAA in plants and inhibits the growth of their root meristematic tissues (Yuan and Huang, 2016; Fattorini et al., 2017; Zhang et al., 2018). Exogenous addition of IAA can alleviate Cd toxicity in plants by reducing Cd uptake and enhancing antioxidant activity (Agami and Mohamed, 2013; Singh and Prasad, 2015). The expression of five ethylene receptors (*ETR*) and the ethylene-responsive transcription factor (*ERF1*) was up regulated in *L. chuanxiong* under Cd stress, indicating the ethylene signaling pathway was active under Cd stress. High concentrations of Cd caused rapid ethylene synthesis in plants, inhibiting cell proliferation in the meristematic tissue zone and cell elongation in the elongation zone of plant roots to hinder root growth (Růzicka et al., 2007; Schellingen et al., 2015; Qin and Huang, 2018; Wakeel et al., 2018). Salicylic acid (SA) is a ubiquitous plant phenolic compound, and SA pretreatment can enhance the plant antioxidant system, improve photosynthetic capacity, and regulate the uptake and distribution of Cd in plants, thus reducing the inhibition of plant root growth caused by Cd toxicity (Guo et al., 2007; Liu et al., 2016; Emamverdian et al., 2020; Zheng et al., 2022). TGA is a key factor in the SA signaling pathway and determines the downstream responses. In the transcriptome sequencing results of the *L. chuanxiong*, five *TGA family* SA transcription factors were significantly down regulated, which may be the mechanism of the involvement of the SA signaling pathway in the regulation of growth and Cd accumulation in *L. chuanxiong* under Cd stress. Expression of the gibberellin (GA) receptor *GID1* was up regulated under Cd stress. GA was able to affect the division and expansion of plant cells under Cd stress, increase the growth of radicle cells in meristematic tissues, and increase the germination rate and shoot and root length of plants affected by stress (Emamverdian et al., 2020). For example, GA application improved root morphology and vigor of lettuce under Cd treatment and attenuated the toxic effects of Cd on lettuce leaves by reducing root uptake of Cd and root crown translocation of Cd (Chen et al., 2021a). In addition, DEGs of the GA signaling pathway (*GH3* and *JAR1*) and abscisic acid (ABA) signaling pathway (*PYL* and *PP2C*) were also regulated by Cd. Exogenous application of jasmonic acid (JA) helped enhance rice’s antioxidant capacity, promoted the chelation of Cd with soluble pectin in rice roots, increased cell wall compartmentalization of Cd, and inhibited the transfer of Cd to protoplasts, thus reducing Cd uptake in roots and aboveground parts (Singh and Shah, 2014; Li et al., 2022). Abscisic acid (ABA) has also been shown to reduce Cd uptake in strawberries effectively (Kocaman, 2023). In conclusion, various phytohormone signaling pathways play important roles in plant responses to Cd stress. These pathways regulate plant growth, Cd accumulation, and antioxidant activity, and their modulation (e.g., application of exogenous hormones) may alleviate the Cd toxicity of *L. chuanxiong*.

### 4.6 Cd treatment may enhance the cell wall structure of the root system of *L. chuanxiong*, in order to improve the plant’s tolerance to Cd

The cell wall is composed of cellulose, hemicellulose, pectin, and lignin (Cosgrove, 2005), and it plays a crucial role in plant defense against Cd toxicity. By limiting the transport of Cd across the cell membrane, the cell wall can reduce the Cd concentration in the protoplasts and protect plants from toxicity (Krzesłowska, 2011; Le Gall et al., 2015). The plant cell wall modification pathway showed upregulation of seven DEGs associated with pectin esterase synthesis. The increased activity of pectin esterase (PME) leads to higher levels of de-esterified pectin, resulting in increased negative charges available to bind Cd^2+^ ions (Xiong et al., 2009). For instance, a study on alfalfa seedlings demonstrated that aeration of the root system increased pectin content and PME activity, ultimately reducing Cd toxicity (Zhao et al., 2019b). In our study with *L. chuanxiong* seedlings, we observed upregulation of the cellulose synthase (CESA) gene involved in cellulose synthesis after Cd stress exposure. Similar results were found by Guo et al. (2016) in their investigation of *Miscanthus sacchariflorus.* CESA is a key enzyme involved in plant cell wall biosynthesis. Previous studies have shown that interference with CESA, such as in the rice *CESA9* mutant created by Song et al. (2013), can reduce Cd accumulation in plants. Furthermore, lignin biosynthesis plays a role in Cd accumulation in plants (Finger-Teixeira et al., 2010). In our transcriptome sequencing results of *L. chuanxiong*, we observed upregulation of genes involved in lignin biosynthesis, including laccase, caffeic acid-O-methyltransferase, bergaptol *O*-methyltransferase, and cinnamaldehyde dehydrogenase cinnamyl alcohol dehydrogenase, in the roots of *L. chuanxiong*. Under Cd stress, lignin content in plant cell walls increased and cell wall structure was enhanced, resulting in reduced Cd penetration and enhanced plant tolerance to Cd (Rui et al., 2016; Zhou et al., 2016). Increased lignin content also enhances the lignification of the endodermis of plant roots, which can effectively prevent a higher proportion of Cd from entering the xylem through the apoplast, thus limiting Cd transport to the aboveground parts (Feng et al., 2018). To summarize, the cell wall components, including pectin esterase, cellulose synthase, and lignin, play crucial roles in plant defense against Cd toxicity. Enhancing their activities and expression levels can improve Cd tolerance. Further research in this area is warranted to deepen our understanding of the mechanisms underlying plant responses to Cd stress.

### 4.7 Metal transporter proteins play an important role in Cd transport and accumulation in *L. chuanxiong*

Heavy metal transporters are essential for the transport and accumulation of heavy metals in plants (Fan et al., 2023). Research has found that some metal transporters in the ZIP family, NRAMP family, and ABC transporter family in plants have the function of transporting Cd (Sterckeman and Thomine, 2020; Fan et al., 2023). For example, the NRAMP family can bind and transport Mn^2+^, Fe^2+^, and Zn^2+^ and participate in the absorption of Cd^2+^ by plant cells and the transport of Cd/Cd complexes (Oomen et al., 2009; Sasaki et al., 2012; Ehrnstorfer et al., 2014). The IRT1 of the ZIP family can transport various metal ions such as Cd^2+^, Zn^2+^, Mn^2+^, and Fe^2+^ (Guerinot, 2000; Long et al., 2018; Halimaa et al., 2019). Some Ca^2+^ transporter proteins in the cell membrane have been shown to have the ability to transport Cd^2+^ (Kabała and Kłobus, 2005; Demidchik et al., 2018; Rivetta et al., 2023). In the RNA-seq results, we annotated the upregulated expression of several Ca transporter protein-related genes (*ATPase4* and *ATPase13*), and they may be involved in the endocytic flow of Cd in *L. chuanxiong* cells. The ABC superfamily (ABC), one of the largest and most diverse protein families in plants, actively participates in the active transport of metal chelates between biological membranes (Fan et al., 2023). The ABCB and ABCC protein subfamilies of the ABC transporter protein family, which are usually localized to the tonoplast, transport glutathione-Cd and phytochelatin-Cd complexes from the cytoplasm to the vacuole for sequestration, respectively, to enhance the tolerance of plants to Cd (Ortiz et al., 1992; Ortiz et al., 1995; Fu et al., 2017). In our results, several genes of *ABCB1* and *ABCC2* were significantly upregulated, and they played an important role in intravesicular transport and sequestration of Cd in *L. chuanxiong*, as well as a key factor in enhancing Cd tolerance and the accumulation capacity of *L. chuanxiong.* In addition to the ABC transporter family, a gene related to the Cd/Zn transporter (*HMA3*) has also been upregulated. *HMA3* is located on the tonoplast and has been shown to have the function of transporting Cd, which may also be involved in the transfer of Cd from the cytoplasm of *L. chuanxiong* to the vacuoles (Ueno et al., 2011; Sasaki et al., 2014). ABCG protein is one of the few known Cd efflux proteins that can eliminate Cd from root epidermal cells and reduce Cd toxicity in plants. For example, overexpression of the *PtoABCG36* gene reduced Cd accumulation in *Arabidopsis* (Wang et al., 2019a). In our results, two ABCG protein subfamily genes were differentially expressed, and they may play an important role in Cd efflux in *L. chuanxiong.* The above findings highlight the critical roles of multiple metal transporter proteins in Cd transport and accumulation in *L. chuanxiong*. These findings help to deepen our understanding of the mechanisms of Cd tolerance and accumulation in *L. chuanxiong*.

## 5 Conclusions

This study analyzed the growth characteristics, physiological indices, and transcriptomic data of *L. chuanxiong* under different concentrations of Cd treatment. We explored the effects of Cd stress on the growth and development of *L. chuanxiong* seedlings and proposed insights into Cd accumulation and tolerance mechanisms in *L. chuanxiong*. The decrease in photosynthetic capacity and the regulation of plant hormone levels appear to be major factors contributing to growth inhibition in response to Cd stress. Moreover, the upregulation of differential genes involved in cell wall modification suggests a potential mechanism for enhancing root cell wall capabilities in isolating and sequestering Cd. The involvement of specific metal transport proteins further highlights their importance in Cd movement within the plant. This study enhances our understanding of the impacts of Cd stress on *L. chuanxiong* and provides a basis for future research in this field.

## Data availability statement

The data presented in the study are deposited in the National Center for Biotechnology Information Biology Project (NCBI) repository, accession number PRJNA960886.

## Author contributions

ZZ: Conceptualization, Data curation, Formal Analysis, Methodology, Resources, Software, Validation, Writing – original draft. LZ: Conceptualization, Data curation, Funding acquisition, Project administration, Writing – original draft. WX: Formal Analysis, Investigation, Methodology, Software, Writing – review & editing. YD: Formal Analysis, Investigation, Software, Writing – review & editing. GH: Methodology, Resources, Supervision, Writing – review & editing. ZY: Methodology, Resources, Conceptualization, Writing – review & editing. DH: Conceptualization, Funding acquisition, Project administration, Supervision, Writing – review & editing. CZ: Funding acquisition, Project administration, Resources, Investigation, Writing – review & editing.
