# Supplementary material for: Transcriptomics combined with physiological analysis reveals the mechanism of cadmium uptake and tolerance in Ligusticum chuanxiong Hort. under cadmium treatment
Source: Front Plant Sci. 2023 Sep 22;14:1263981. doi: 10.3389/fpls.2023.1263981 (PMC10556529; doi:10.3389/fpls.2023.1263981)
Supplement: Supplementary file 2 [file Table_2.docx]

## Methods for calculating the activities of POD, SOD, and CAT enzymes, as well as the determination of MDA and GSH contents.

Follow the instructions in the manual to process the samples. Add the processed sample solution to a 96-well plate and measure the absorbance at specific wavelengths using an enzyme-linked immunosorbent assay (ELISA) reader. Then, calculate the enzyme activity or content using the formulas provided.

## CAT

### Measurement of absorbance:

Record the initial absorbance value (A1) at 240 nm and the absorbance value (A2) after 1 minute. Calculate ΔA = A1 - A2.

### Definition and calculation formula of the unit:

**Unit definition:**

One enzyme activity unit is defined as the degradation of 1 nmol H_2_O_2_ per minute per gram of tissue.

**Calculation formula:**

CAT (nmol/min/g fresh weight) = [ΔA × V_total_ ÷ (ε × d) × 10^9^] ÷ (W × V_sample_ ÷ V_total sample_) ÷ T = 918 × ΔA ÷ W

**V_total_:** Total volume of the reaction system, 2 × 10^-4^ L;

**ε:** Molar extinction coefficient of H_2_O_2_, 4.36 × 10^4^ L/mol/cm;

**d:** Optical path length of the 96-well plate, 0.5 cm;

**V_sample_:** Volume of the sample added, 0.01 ml;

**V_total sample_:** Volume of the extraction solution added, 1 ml;

**T:** Reaction time, 1 min;

**W:** Sample mass, g.

## POD

### Measurement of absorbance:

Record the absorbance value (A1) at 470 nm after 1 minute and the absorbance value (A2) after 2 minutes. Calculate ΔA = A2 - A1.

### Definition and calculation formula of the unit:

**Unit definition:**

One enzyme activity unit is defined as a change of 0.005 A470 per minute per gram of tissue in a reaction system with a volume of 1 ml.

**Calculation formula:**

POD (U/g fresh weight): =ΔA × V_total_ ÷ (W × V_sample_ ÷ V_total sample_) ÷ 0.005 ÷ T = 4000 × ΔA ÷ W.

**V_total_:** Total volume of the reaction system, 0.2 ml;

**V_sample_:** Volume of the sample added, 0.01 ml;

**V_total sample_:** Volume of the extraction solution added, 1 ml;

**T:** Reaction time, 1 minute;

**W:** Sample mass, g.

## SOD

### Measurement of absorbance:

Add the sample solution to a 96-well plate, mix well, let it stand at room temperature for 30 minutes, and then measure the absorbance at 560 nm.

### Definition and calculation formula of the unit:

**Unit definition:**

One unit of SOD enzyme activity is defined as the percentage of inhibition when the reaction system inhibits 50% in the above-mentioned xanthine oxidase-coupled reaction system (U/ml).

**Calculation of inhibition percentage:**

Inhibition percentage = (A_control_ - A_blank_) ÷ A_control_ × 100%

**Calculation formula:**

SOD activity (U/g fresh weight): = [Inhibition percentage ÷ (1 - Inhibition percentage) × V_total_] ÷ (W × V_sample_ ÷ V_total sample_) = 11.11 × Inhibition percentage ÷ (1 - Inhibition percentage) ÷ W

**V_total_:** Total volume of the reaction system, 0.2 ml;

**V_sample_:** Volume of the sample added, 0.018 ml;

**V_total sample_:** Volume of the extraction solution added, 1 ml;

**W:** Sample mass, g.

## MDA

## Measurement of absorbance:

Measure the absorbance at 532 nm and 600 nm, and calculate ΔA = A532 - A600.

**Calculation formula:**

MDA (nmol/g fresh weight): = [ΔA × V_total_ ÷ (ε × d) × 10^9^] ÷ (W × V_sample_ ÷ V_total sample_) = 516 × ΔA ÷ W

**V_total_:** Total volume of the reaction system, 4 × 10^-4^ L;

**ε:** Molar extinction coefficient of MDA, 1.55 × 10^3^ L/mol/cm;

**d:** Optical path length of the 96-well plate, 0.5 cm;

**V_sample_:** Volume of the sample added, 0.1 ml;

**V_total sample_:** Volume of the extraction solution added, 1 ml;

**W:** Sample mass, g.

## GSH

### Measurement of absorbance:

Measure the absorbance of the blank and test samples at 412 nm, and record them as A1 and A2, respectively.

### The formula for the GSH standard curve:

y = 0.75x (x is the GSH concentration in μmol/ml, y is the absorbance value).

**Calculation formula:**

GSH (μmol/g fresh weight) = (A2 - A1) ÷ 0.75 × V_sample_ ÷ (V_sample_ ÷ V_total sample_ × W) = 1.334 × (A2 - A1) ÷ W

**V_sample_:** Volume of the supernatant added to the reaction system, 20 μL;

**V_total sample_:** Total volume of the supernatant, 1 ml;

**W:** Sample weight, g.
